# Recent advancements in the exploitation of the gut microbiome in the diagnosis and treatment of colorectal cancer

**DOI:** 10.1042/BSR20204113

**Published:** 2021-07-26

**Authors:** Katie J. Stott, Bethan Phillips, Lee Parry, Stephanie May

**Affiliations:** 1 European Cancer Stem Cell Research Institute, School of Biosciences, Cardiff University, Cardiff CF24 4HQ, U.K.; 2 CRUK Beatson Institute, Garscube Estate, Switchback Road, Bearsden, Glasgow G61 1BD, U.K.

**Keywords:** bacterio-thearpy, colorectal cancer, diagnostic biomarkers, intestine, microbiome

## Abstract

Over the last few decades it has been established that the complex interaction between the host and the multitude of organisms that compose the intestinal microbiota plays an important role in human metabolic health and disease. Whilst there is no defined consensus on the composition of a healthy microbiome due to confounding factors such as ethnicity, geographical locations, age and sex, there are undoubtably populations of microbes that are consistently dysregulated in gut diseases including colorectal cancer (CRC). In this review, we discuss the most recent advances in the application of the gut microbiota, not just bacteria, and derived microbial compounds in the diagnosis of CRC and the potential to exploit microbes as novel agents in the management and treatment of CRC. We highlight examples of the microbiota, and their derivatives, that have the potential to become standalone diagnostic tools or be used in combination with current screening techniques to improve sensitivity and specificity for earlier CRC diagnoses and provide a perspective on their potential as biotherapeutics with translatability to clinical trials.

## Introduction

Over recent years’ research into the human microbiome, a collection of interacting microbes including bacteria, viruses, fungi, archaea, protozoa and helminths that reside within and on the human body, has demonstrated mechanistic roles in the maintenance of health and roles in disease [1,2]. The majority of these microorganisms are commensal and populate the intestines where they have important roles in the digestion of food, providing energy sources for colonocytes (through the production of short-chain fatty acids (SCFAs) such as butyrate), modulating and training the host immunity, protecting from pathogens and pathogen overgrowth, maintenance of host-tissue structure and proliferation, regulating endocrine function, neurological signalling, aiding metabolism, eliminating toxins, and modifying drug actions. Microbiome imbalance, known as dysbiosis, with regard to the composition and/or function of the microbiome is widely associated with the onset of several intestinal pathologies including inflammatory bowel disease [3,4], ulcerative colitis [5], Crohn’s disease [5] and colorectal cancer (CRC) [6,7]; and extraintestinal diseases such as obesity [8], type 2 diabetes [9], cardiovascular disease [10], non-alcoholic liver disease [11] and non-alcoholic steatohepatitis [1,12]. There are a range of endogenous and exogenous factors that can manipulate the gut microbiome such as mode of birth, host genetics and immune responses, biological sex, age, diet and lifestyle (supplements, breastfeeding, lack of fibre, fruit and vegetables in the diet and lack of physical activity), medication and xenobiotics (including antibiotics), infections and exposures to other environmental microbes. Many of these factors are associated with an altered risk of developing cancer, in particular CRC, on which the microbiome is known to impact CRC development and progression [1,2].

**Figure 1:**
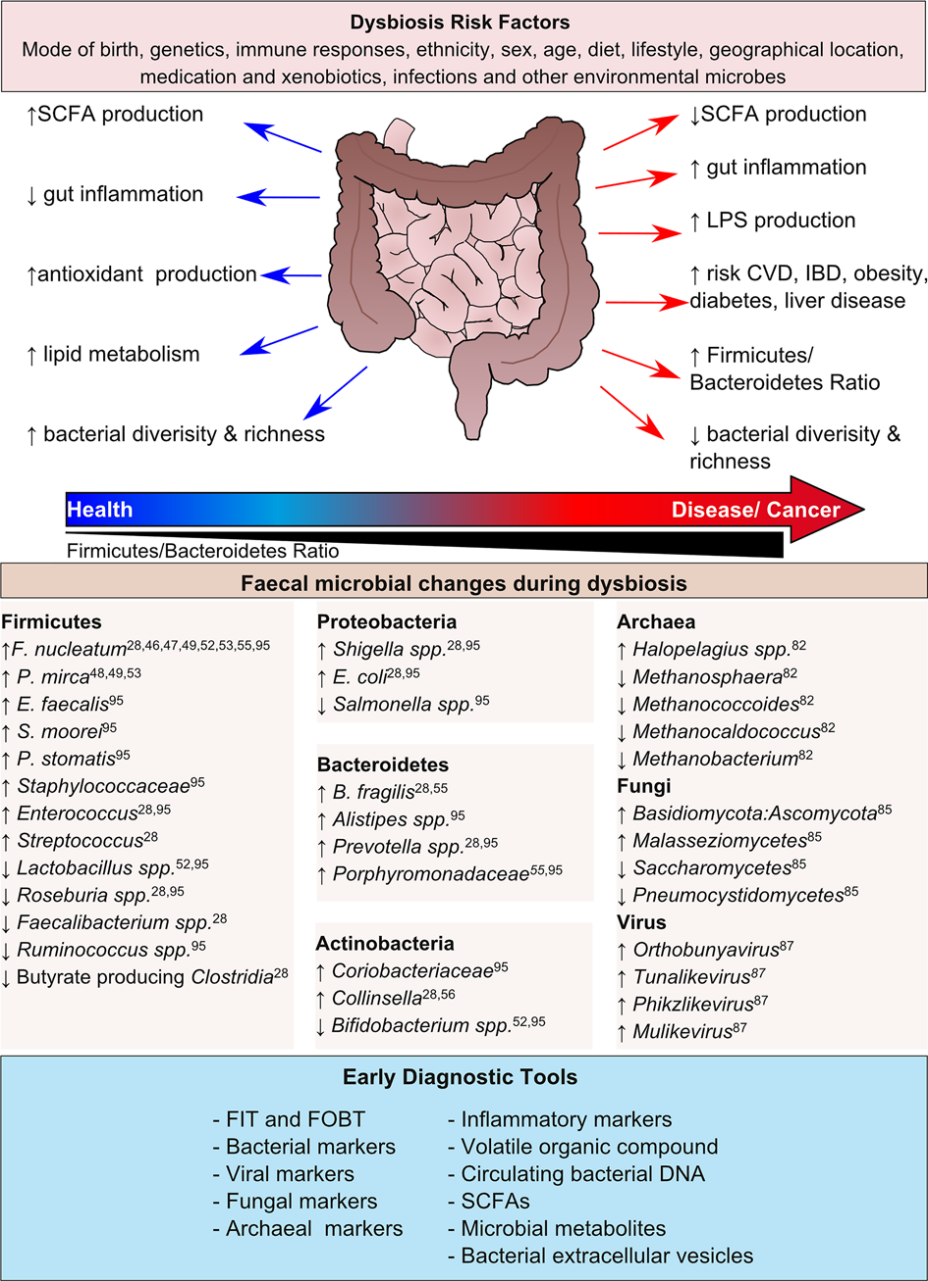
Schematic representation of the transitioning role of the gut microbiota from healthy to diseased states with examples of microbial changes and diagnostics techniques

The different types of intestinal microbes, their composition and mechanistic involvement in human health and disease have previously been comprehensively reviewed [1]. At the forefront of research today is whether we can exploit aspects of the gut microbiota for the detection and treatment of cancer. In this review, we will discuss the latest advances in utilising the microbiome as a diagnostic tool and how we can exploit microbes as therapeutic agents in the treatment and management of CRC (Figure 1).

### Microbiome in the healthy gut

When defining what a healthy microbiome looks like we are faced with several challenges, due to the vast differences in microbial compositions among seemingly healthy individuals. As such, a healthy microbiome in one person does not always represent a healthy microbiome in another person and could in fact be undistinguishable from an unhealthy microbiome in a third person. As previously alluded to, differences in host genetics, environmental and lifestyle factors including a person’s ethnicity, geographical location and urbanisation [13–15] can have subtle but substantial consequences on altering the gut microbiome, which can increase the host’s risk of developing disease. However, studies have shown that a healthy person’s microbiome and associated metabolome is relatively stable over time [16,17] and thus longitudinal surveillance within individuals could serve as a useful diagnostic and therapeutic tool. In general a healthy microbiome is characterised by having a high taxa diversity and microbial gene richness and a stable functional microbial core [16]. Stable microbial core functions include genes that encode glycosaminoglycan degradation, the production of SCFAs such as butyrate, propionate and acetate) via fermentation of polysaccharides and the biosynthesis of compounds used by the host such as production of essential amino acids and vitamins [18].

The major constituent of the gut microbiota are bacteria and taxonomically they are classified according to phyla, classes, orders, families, genera and species. Accounting for approx. 90% of the gut microbiota, the dominant bacteria phyla are Firmicutes and Bacteroidetes followed by Actinobacteria, Proteobacteria, Fusobacteria and Verrucomicrobia [19,20]. Examples of genera within the Firmicutes phylum are *Lactobacillus, Bacillus, Clostridium, Enterococcus* and *Ruminococcus.* The Bacteroidetes phylum consists predominantly of *Bacteroides* and *Prevotella*. The *Bifidobacterium* genus is a major constituent of the Actinobacteria phylum although this phylum is significantly less abundant in comparison to Firmicutes and Bacteroidetes phyla [19]. Having to adapt to acute stresses and changes in dietary components, the gut microbiota is quite plastic in its composition, but chronic perturbation of the microbes leading to dysbiosis through selection of more virulent opportunistic microorganisms is widely associated with the onset of several diseases, and as such the microbial changes related to such disorders have been extensively documented in recent years.

### Microbial dysbiosis

While gut dysbiosis is most commonly associated with pathological conditions of the gastrointestinal tract (GI), it can also impact upon other systems. This includes conditions of the immune system, the central nervous system and alterations to the host’s metabolism [1,21]. The role of gut dysbiosis in some of these metabolic pathologies is reviewed in detail by Fan and Pedersen (2021) [1].

A common marker of gut dysbiosis and pathological disorders is the relationship between the Firmicutes and Bacteroidetes phyla, termed the Firmicutes/Bacteroidetes ratio. This was first noted in murine studies using obese mice and lean controls, which demonstrated that obese mice had an increased load of Firmicutes at the expense of Bacteroidetes (50% reduction) [22]. Further to this, it was shown that obese patients also had an increased Firmicutes/Bacteroidetes ratio compared with lean healthy controls, but when started on a diet therapy (fat-restricted or a carbohydrate-restricted low-calorie diet) over 1 year the relative abundance of Bacteroidetes increased whilst the abundance of Firmicutes decreased in combination with weight loss over time [23]. Interestingly, despite vast amounts of evidence of an altered Firmicutes/Bacteroidetes ratio in disease, several studies have failed to detect Firmicutes/Bacteroidetes ratio alterations, possibly due to differences in ages, ethnicity and sex but instead report diminished bacterial diversities, which may be another indicator of disease [24–26]. Specifically in the context of CRC, it has recently been shown that the bacterial diversity and richness is reduced, compared with healthy individuals, in murine CRC models and CRC patient intestinal mucosa and faecal samples [7,27]; with particular alterations to populations of bacteria that can modulate the mucosal immune response [27] and reductions in healthy probiotic bacteria [7]. Through analysis of faecal samples from CRC patients and healthy individuals, the abundance of Bacteroidetes was significantly reduced while Proteobacteria were more abundant in CRC patients compared with healthy controls. When looking at the microbial alterations at the genus level specifically within CRC patients, the presence of the following have been reported; Firmicutes (*Fusobacterium, Gemella, Faecalibacterium, Blautia, Enterococcus, Subdoligranulum, Dorea, Megamonas* and *Streptococcus)*, Bacteroidetes (*Bacteroides, Prevotella, Parabacteroides* and *Porphyromonas)*, Actinobacteria *(Bifidobacterium* and *Collinsella)* and Proteobacteria (*Escherichia/Shigella*). The relative abundances of the following genera were significantly depleted in CRC patients compared with healthy controls; *Alistipes, Phascolarctobacterium, Oscillibacter, Bacteroides, Roseburia, Eubacterium, Parasutterella* and unclassified genera of the order *Clostridiales* [28].

However, the present study does not distinguish between tumour-associated microbes and global CRC microbiome alterations. In another CRC patient study by Ahn et al. (2013) a decrease in bacterial diversity, in particular *Clostridia* (Firmicutes) microbes, was reported from faecal samples, and an increase in *Fusobacterium nucleatum* and *Porphyromonas* [29]. Both these studies highlight a decrease in bacteria that ferment dietary fibre to SCFAs in the colon. The production of SCFAs, in particular butyrate which is the primary energy source of colonocytes and fuels the weekly replacement of the epithelial barrier, is essential to the homoeostatic maintenance of the gut. A lack of butyrate-producing bacteria can have detrimental consequences in disease progression but also highlights the potential of SCFA and microbe-derived metabolites as biomarkers and diagnostic tools for CRC [30–32].

## The gut microbiome as a novel biomarker for CRC diagnosis

Early diagnosis of CRC correlates with better prognosis, with survival exceeding 90% in cases that are diagnosed and treated at an early and localised stage (stages I and II) [33,34] which significantly decreases to 13% in patients diagnosed with advanced metastatic disease [35]. This combined with the long latency period of the transition from adenoma to carcinoma [36] makes CRC a suitable target for population screening, which has been implemented in many developed countries.

Colonoscopy is considered the gold standard for CRC diagnosis, and the American Cancer Society recommends a colonoscopy every 10 years from the age of 45 [37]. Despite this, colonoscopy is not often the primary test for early CRC diagnosis due to its invasive nature. Current screening programmes commonly utilise either faecal occult blood tests (FOBTs) or faecal immunochemical test (FIT), where the latter has overtaken the former due to increased sensitivity and uptake [37]. Individuals with positive results are referred to for follow-up colonoscopies, where any polyps can often be removed simultaneously and sent for histological diagnosis. However, FOBTs and FIT have been associated with a high rate of false-positive results [38,39] causing an unnecessary burden on patients and healthcare systems. Additionally, both have been shown to have low sensitivity to pre-cancerous lesions and colorectal adenomas (CRAs) [40].

### Bacterial diagnostic markers

Multiple species of microbes have been shown to be significantly altered in patients with CRC compared with healthy controls [41]. These significantly altered microbes may possess non-invasive diagnostic potential, which may aid better identification of individuals who require a colonoscopy. Research has begun to focus on developing diagnostic models based on microbial markers associated with CRC. Due to the non-invasive nature, the majority of studies have focused on bacterial biomarkers from stool samples, but progress is also being made using blood and urine.

#### Bacterial diagnostic markers in stool

Despite an increasingly large number of studies that utilise the microbiome for CRC diagnostic purposes, there is yet to be a clear consensus on suitable biomarkers. The main reason for this is the large variation between studies. It is well known that many external variables can alter the gut microbiome, which may well be part of the reason that a consensus is yet to be reached. Given that the microbiome can be altered by many external factors, such as diet [42,43], sex [43], age [43] and antibiotic exposure [44], it is not surprising that many studies have shown sample variation [43,45–47]. One study identified a diagnostic model of 22 biomarker genes whose abundances were altered between CRC patients and healthy family member controls from Chongqing, China, and applied it to published metagenomics data from two different geographical locations, Hong Kong and France [48]. Here they found that the Chinese biomarker panel had a reduced diagnostic capability in these locations, but report reproducible region-specific diagnoses [48]. This highlights the importance of collecting region-specific microbiome data and the potential to exploit such datasets in the diagnoses of their respective regional diseases.

A variety of approaches are being considered to improve the diagnostic specificity of stool analysis, with differences in the bacterial markers utilised, the number of bacterial markers used and the relative abundances of bacteria and their metabolites.

##### Faecal bacterial markers

One such bacterium being widely considered to complement current FITs is *Fusobacterium nucleatum. F. nucleatum* is one of the most commonly identified bacteria in CRC patients and potentially reflects tumour location as *F. nucleatum* is more commonoly associated with left-side colon cancer compared with the right [49]. Additionally, *F. nucleatum* has been shown to promote tumorigenesis and chemoresistance in CRC and has been associated with a poorer overall survival rate in advanced CRC patients [50]. However, several other bacterial species have shown to be significantly altered in CRC (summarised in Gagnière et al., [41]) and have been assessed for CRC biomarker potential, e.g. *Parvimonas micra* [51].

One study by Wong et al., (2017) investigated the potential of *F. nucleatum*, in addition to *Peptostreptococcus anaerobes* and *P. micra* [52], all previously found to be increased in CRC [53,54]. The relative abundances were measured from stool samples using quantitative polymerase chain reaction (qPCR). All three markers showed significantly higher abundance in patients with CRC compared with control, and *F. nucleatum* was also significantly more abundant in patients with advanced adenoma. When combined with FIT, the *F. nucleatum* biomarker showed superior sensitivity than FIT alone in detecting CRC, and additionally increased the performance of adenoma detection, emphasising the potential of bacterial biomarkers as more useful diagnostic tools over current diagnostic strategies [52].

In contrast with using a single biomarker, Guo et al., (2018) evaluated whether a ratio of *F. nucleatum* to probiotic populations (*Lactobacillus, Bifidobacterium* and *Faecalibacterium prausnitzii*), in which the latter has been shown to be reduced in CRC (probiotics are discussed later on in this review), could be a useful diagnostic biomarker for CRC [55]. The ratio *F. nucleatum/Bifidobacterium* showed superior sensitivity of 84.6% and specificity of 92.3% for diagnosing CRC, versus 46.2% sensitivity using *F. nucleatum* alone. Whilst the ratio showed an area under the curve (AUC) value of 0.876 for detecting early stages of CRC (stages I and II), a limitation of this study was that patients with adenomas were not investigated. The ability to detect CRAs is essential for an early diagnostic tool. Additionally, the ratio could be further used in accordance with FIT [55].

Whilst there have been numerous studies that have primarily focused on bacterial abundances as novel diagnostic tools [56,57], some of which have solely focused on adenoma detection [58], various other microbial-derived markers have been identified (Table 1).

**Table 1:** Validation studies of microbial biomarkers for diagnosing CRC

Sample matrix	Biomarker(s) studied	Detection method	Diagnostic performance	Reference
Stool	*P. micra*	qPCR	*P. micra* accurately discriminated CRC patients from healthy controls. It has poorer performance discriminating CRA from controls	[51]
Stool	*F. nucleatum, P. anaerobius* and *P. micra*	qPCR	*F. nucleatum* alone showed superior performance to detect CRC compared with FIT. Combination of FIT and *F. nucleatum* increased performance of FIT for both CRC and CRA detection	[52]
Stool	*F. nucleatum (Fn), F. prausnitzii, Bifidobacterium (Bb)* and *Lactobacillus*	qPCR	*Fn/Bb* ratio had superior sensitivity and specificity to detect CRC compared with *Fn* alone and other ratios investigated	[55]
Stool	*P. micra, F. nucleatum and clba +* bacteria	qPCR	*P. micra* alone could detect CRC with specificity of 87.3% and sensitivity of 60.5%. Combination of *P. micra* with the other markers or FIT increased sensitivity, but decreased specificity	[56]
Stool	*F. nucleatum, E. feacalis, S. bovis, Enterotoxigenic Bacteroides fragilis (ETBF)* and *Porphyromonas spp.*	qPCR	Using a combination of all microbial markers gave the best diagnostic performance compared with each marker alone. The combined model could discriminate both adenomatous polyps and CRC patients from healthy controls for early detection with sensitivity and specificity up to 93.1 and 93.5% respectively	[58]
Stool (extracellular vesicles)	Two bacterial genera (*Collinsella* and *Solanum*) and two metabolites (leucince and oxalic acid)	GC-TOF-MS and NGS	Bacterial AUC, sensitivity and specificity for predicting CRC were 95, 90 and 100%, respectively Metabolite AUC, sensitivity and specificity for predicting CRC were 92, 80 and 100% respectively When used in combination the dual panel had an AUC of 100% for predicting CRC in patients	[59]
Stool	23 metabolites	UPLC-MS/MS	Whilst all metabolites were significantly altered in patients with CRC and CRA, the changes were too small to be used for diagnostic purposes. Additionally, many of the changes were weaker in the CRC group	[46]
Stool	SCFAs: acetate, propionate and butyrate	HPLC	When incorporated into a bacterial gene-based diagnostic model, the SCFAs failed to increase performance	[60]
Oral swab	*Fusobacterium, Cyanobacteria* [unclassified], *Veillonella, Selenomonas* and *Gemella*	NGS	A random forest model using the five markers could accurately distinguish between CRC patients and healthy controls	[61]
Blood	LPS, lipid metabolism, inflammatory markers and vascular damage markers	Blood pathology testing and TEG	LPS levels and inflammatory (e.g. SAA and CRP) and hypercoagulability markers were elevated in CRC patients	[62]
Blood	Circulating bacterial DNA; *E. rectale, B. adolescentis, R. torques, R. intestinalis* and *P. freudenreichii*	WGS	Twenty-eight bacterial species from circulating bacterial DNA had superior ability to differentiate between healthy individuals and CRC/CRA patients	[63]
Urine	VOC profiles	LC‐FAIMS‐MS	The VOC profiles of CRC were able to be distinguished from healthy controls with an AUC value of 0.72	[64]

Abbreviations: CRP, C-reactive protein; GC-TOF-MS, gas chromatography/time-of-flight mass spectrometry; HPLC, high performance liquid chromatography; LC‐FAIMS‐MS, liquid chromatography-high field asymmetric waveform ion mobility spectrometry-tandem mass spectrometry; LPS, lipopolysaccharide; NGS, next-generation sequencing; SAA, serum amyloid A; TEG, thromboelastography; UPLC-MS/MS, ultraperformance liquid chromatography-tandem mass spectrometry; VOC, volatile organic compound; WGS, whole-genome sequencing.

##### Faecal bacterial metabolites

What constitutes a healthy or cancer microbiome varies so greatly among individuals that the likelihood of individual bacterial strains being diagnostically effective in all populations and at all levels is extremely unlikely. To overcome this, researchers have focussed on the metabolite output of the microbiome rather than its component strains. Many effects of the microbiome on CRC development have been directly associated with the bacterial metabolome, rather than the microbiota itself, such as the production of SCFAs [65]. For example SCFAs, produced by fibre-fermenting bacteria in the gut have potent anti-inflammatory properties, provide the major fuel source for colonocytes, impact on endocrine functions and are viewed as potent chemopreventatives [66]. However, concentrations of the SCFAs: acetate, propionate and butyrate measured by high-performance liquid chromatography, showed no significant associations with tumour status, or between CRC patients and healthy controls. When incorporated into a random forest classifier, SCFAs failed to improve the performance of bacterial gene-based diagnostic models and thus argues against the use of faecal SCFAs as a biomarker to diagnose a patient with an adenoma or carcinoma [60]. However, it is worthy to note that these samples were taken at a single point in time and thus may not reflect changes or associations of SCFA concentration with tumour progression over time. A different study investigated the global metabolomic profiles of stool samples of patients with advanced adenomas and CRCs in comparison to matched controls. Despite identifying 24 metabolites (particularly lipids) that were differentially altered in CRA patients compared with controls, with similar trends seen in the CRC group, the changes were too small to be used for diagnostic purposes. Many of the differences in metabolites were also found to be weaker in the CRC group compared with CRA, and no metabolite seemed to increase along the adenoma-carcinoma sequence, but these data do give an insight into some of potential metabolic changes that occur in the earlier stages of CRC progression [46]. Additionally, this study also identified sex-dependent metabolite alterations [46] and as such highlights the importance of considering sex in biomarker identification studies and treatment regimens for CRC patients as well as determining the role of other factors such as ethnicity on the microbiome and metabolome.

##### Secreted bacterial biomarkers

Alternative approaches have looked at metabolite abundance in bacterial extracellular vesicles (BEVs). Alterations in BEVs are associated with many conditions [67,68] including allergies and neutrophilic pulmonary inflammation caused by environmental exposure to *Staphylococcus aureus*-derived EVs found in household dust particles [69]. It is common for BEVs to contain microbe-associated or pathogen-associated molecular patterns (MAMPs/PAMPs; such as lipopolysaccharide (LPS) and peptidoglycan) that facilities the interaction with the immune system via Toll-like receptors (TLRs). Depending on the type of immune cell, activation of TLR signalling can lead to a range of immune responses [70]. Given the ability of BEVs to influence host immune cell signalling BEVs may have untapped diagnostic and therapeutic potential. Thus they offer potential as biomarkers due to their presence and as indicators of alterations to microbial metabolites due to CRC bacterial dysbiosis [59]. It has previously been shown that BEVs constitute approx. a quarter of the total bacterial DNA isolated from stool samples. As such, findings from the majority of faecal microbial studies are confounded by the inability to differentiate between bacterial DNA and that of BEVs [71]. To take into consideration this potential bias, and to investigate the composition of secreted BEVs and the role they play in intestinal disease, Kim et al. (2020) set out to investigate the association of metagenomics and metabolomics of gut bacterial-derived EVs of CRC patients and healthy individuals [59]. As a biomarker, Kim et al. identified several altered amino acids, carboxylic acids and fatty acids from BEVs from the stool of the CRC group. Metabolites up-regulated included leucine, alanine, phenol, oxalic acid and hexanoic acid, whereas butanoic acid and aminoisobutyric acid were down-regulated. Metagenomic analysis identified several deregulated bacterial species (34 bacterial genera) in CRC subjects in comparison to healthy controls with increases in the relative abundance of Firmicutes and decreases in Proteobacteria and Tenericutes (reporting similar findings to previously discussed stool-based studies). To determine the suitability of their metagenomic and metabolomic biomarkers to differentiate CRC patients from healthy controls, an optimised diagnostic model comprising two metabolites (leucine and oxalic acid) and two bacterial genera (*Collinsella* and *Solanum melongena*) were tested [59]. The bacterial biomarkers were able to predict CRC with an AUC, sensitivity and specificity of 95, 90 and 100% respectively. While less powerful than the metagenomics model, the two metabolic biomarkers were able to predict CRC with an AUC, sensitivity and specificity of 92, 80 and 100%. Amalgamation of both the metabolic and metagenomic models improved the diagnostic capability to 100% (AUC). The present study highlights that metabolic and metagenomic biomarkers derived from BEVs have the potential to accurately diagnose CRC patients. Currently, research into the effect of the bacterial-derived EVs on CRC initiation and progression is still in its early days.

#### Non-stool bacterial biomarkers

Whilst stool samples are non-invasive, current FIT or FOBT-based screening programmes have relatively low uptake (∼60%) [72,73] emphasising the requirement for non-stool methods. Several studies have aimed to characterise microbial-related biomarkers from urine [64,74,75], blood [62,75] and oral samples [61].

##### Oral biomarkers

Zhang et al., (2020) investigated the association between the oral microbiome and CRC by carrying out 16s rRNA sequencing on oral swab samples from CRC patients, CRA patients and healthy controls. Notably, the oral microbial α diversity was higher in the CRA and CRC cohorts compared with control patients, possibly underpinned by translocation of oral bacteria to the GI tract. Interestingly the diversity was higher in the CRA group compared with CRC patients. Specifically, enrichment of phylum Proteobacteria, Bacteroidetes and Fusobacteria and genera *Fusobacterium, Prevotella* and *Porphyromonas* were reported in the CRA group. Fusobacteria and Bacteroidetes particularly, *Fusobacterium, Prevotella, Porphyromonas* and *Veillonella* were enriched in CRA patients compared with healthy controls, whilst *Streptococcus, Gemella*, and *Megamonas* were significantly lower. Comparisons of CRC patients to healthy controls revealed higher bacterial abundances of phyla Fusobacteria and Bacteroidetes and genera *Fusobacterium, Prevotella* and *Veillonella* [61]. Functional and metabolic analyses revealed that CRA and CRC groups had overrepresented cell motility pathways and reduced carbohydrate metabolisms pathways compared with controls. *Streptococcus* was negatively correlated with cell motility and oncogenic pathways, while *Fusobacterium* and *Prevotella* were positively correlated with oncogenic pathways [61]. Due to the differences in bacterial composition, diversity and function among the CRA, CRC and healthy groups in this study, the oral microbiome could serve as a non-invasive predictor of CRA and CRC.

##### Urinary biomarkers

An alternative screening method for some cancers is the detection of volatile organic compounds (VOCs). VOCs are organic compounds, and are products of metabolic processes, such as fermentation of the hosts diet by the intestinal microbiome [76]. Detection of VOC patterns in adenoma and CRC patients (both invasive and non‐invasive) and their efficacy as disease‐specific gas phase biomarkers, has advanced over recent years. VOC analysis from faecal samples has been investigated previously [77] but due to the low uptake of stool‐based screening programmes such analysis has limited clinical benefit and further emphasises the requirement for alternative biomarker approaches. Several studies have reported that exhaled VOC analysis was sufficient to distinguish CRC patients from healthy controls with more than 75% accuracy [78,79] and similar results were obtained when performing urinary VOC analysis [80,81]. More recently in 2019, a study aimed to characterise the urinary VOC profile of CRC patients with correlation to their respective stool microbiome profiles to determine whether the profiles could distinguish between cancer patients and healthy controls. Unlike previous reports, the present study chose to validate their findings through comparison with spouses/cohabitors and first-degree relatives to determine whether the VOCs were different amongst people with the same risk factors (shared environment exposure and genetic factors) [64]. When compared with relative or spouses individually no difference in VOC profiles to CRC patients were identified. However, combination of the relative and spouse group to form a larger control cohort and increase the power of the study showed that the VOC profiles from healthy controls were distinguishable from CRC patients, with an AUC value of 0.72, and a sensitivity and specificity of 0.69 [64]. Despite this modest cancer-specific VOC profile, this study adds to the accumulating evidence that urinary VOCs have a CRC biomarker potential. As in patients with confirmed cancer, FIT was superior (80% sensitivity) to VOC analysis alone (63% sensitivity). However, when applied to a FIT-negative CRC cohort, the two-step combination of FIT testing and urinary VOC detection increased the sensitivity to 97% [74]. This dual testing platform is comparable with the performance of colonoscopy but with a vast reduction in cost and improved patient experience supporting the use of VOC biomarker in the diagnosis of CRC.

##### Blood biomarkers

Several groups have also set out to identify microbial-related biomarkers from blood samples. Gut dysbiosis contributes to gut barrier dysfunction, enabling the translocation of bacteria into the circulation which can cause further inflammation and promotion of tumorigenesis [82]. Increased intestinal permeability can cause persistent inflammation and hypercoagulability due to the release of pro-inflammatory bacterial products such as LPS [62]. LPS can bind to TLR4 and downstream signalling leads to pro-inflammatory gene expression [83]. One study analysed blood samples to investigate microbial differences between CRC and healthy controls and found that CRC patients have significantly higher circulating levels of LPS, indicating barrier dysfunction. Several other alterations found in CRC patients included altered lipid metabolism, inflammatory markers and vascular damage markers. Further investigation would be required but detection of LPS from blood samples shows promise as a CRC biomarker [62].

A novel proof-of-concept study carried out whole-genome sequencing on plasma to characterise alterations in circulating bacterial DNA in CRC and CRA patients. Once the host genome was removed, the remaining sequences were mapped on to bacterial genomes. Bacterial diversity was found to be higher in healthy controls than CRC and CRA groups and the bulk of the genera identified were bacteria commonly associated with GI and oral tracts. Here, 28 bacterial species from circulating bacterial DNA were identified that had superior ability to distinguish CRC and CRA from healthy controls of which many of these species are enriched in CRC cases, *Eubacterium rectale, Bifidobacterium adolescentis, Ruminococcus torques, Roseburia intestinalis* and *Propionibacterium freudenreichii*. Interestingly, many commonly altered species in CRC stool samples were not identified here, e.g. *P. micra* and *F. nucleatum* [63]. This study also compared their findings with faecal metagenomic studies and found a positive correlation between circulating bacterial DNA and faecal bacterial DNA [84].

### Alternative microbial biomarkers

Despite the majority of studies focusing on bacterial biomarkers for early and late CRC screening, a few studies have recently emerged looking at other aspects of the microbiota, including species of archaea, fungi, protozoa and viruses [1].

In 2020 a study by Coker et al*.*, focussed on altered archaea composition [85]. Archaea are single-celled prokaryotes, which are distinct from bacteria and eukaryotes, such that they lack peptidoglycan and fatty acids [86]. Whilst many archaea are found in extreme environments, some species have been isolated from human skin, gut and oral cavity [87]. The archaea that reside in the human colon are nearly always methanogens, obligate anaerobes, such as *Methaonbrevibacter* and *Methanosphaera* [87]. Due to archaea being comparatively low in abundance in the gut, and that they are mostly unculturable, the use of next-generation sequencing is required to assess the archaeaome. Despite no detectable differences in α diversities between stool samples from CRC patients and healthy people, 28 archaeal species were found to be significantly altered with specific separations between early CRC, advanced CRC and healthy patients. In CRC patients a depletion of methanogens and the enrichment of halophilic archaea were seen. Compared with the control group, there was enrichment of *Halopelagius* species and depletion of methanogens including *Methanosphaera, Methanococcoides, Methanocorpusculum, Methanocaldococcus* and *Methanobacterium* in CRC patients. Species that were depleted in advanced CRC compared with early CRC included *Methanocaldococcus* and *Methanotorris* [85]. Thus an alternative CRC biomarker approach could be the detection of halophilic archaea and depletion of methanogens from stool samples.

The CRC gut fungal microbiota remains largely unknown however, a recent study set out to identify changes in the gut fungal microbiota in CRC [88]. The group identified a CRC-associated faecal fungal dysbiosis signature. Specifically an increased Basidiomycota:Ascomycota ratio, which has been shown to reflect fungal dysbiosis in irritable bowel disorders [89], was observed in patients with CRC compared with healthy controls. There were also significant depletions in Saccharomycetes and Pneumocystidomycetes classes in CRC patients while Malasseziomycetes were enriched. Some of the fungal biomarkers identified include *Aspergillus flavus, Kwoniella mangrovensis* and *Pseudogymnoascus* sp. Additionally, the markers were further validated using independent Chinese and European cohorts [88]. Identification of a fungal dysbiosis in CRC patients suggests that fungal faecal markers could be exploited for diagnostic purposes, but further research into suitable biomarkers covering a range of different risk factors is required to benefit current clinical screening.

Finally, several studies have identified differences in the gut virome between CRC patients and healthy controls. One of the first studies to investigate the faecal virome of CRC patients was by Nakatsu et al., (2018) [90]. Here they identified a relative increase in bacteriophage richness in CRC metagenomes and a significant increase in the diversity of the bacteriophage virome which was associated with a reduced bacterial diversity in CRC patients. They identified 22 viral genera that differentiated CRC patients from controls, including the following taxa *Orthobunyavirus, Tunalikevirus, Phikzlikevirus, Betabaculovirus, Sp6likevirus, Punalikevirus, Lambdalikevirus, C2likevirus* and *Mulikevirus.* To assess the suitability of this discovery virome cohort as a predictor of CRC status, Nakatsu et al. went on to compare their data with three published independent studies to evaluate its potential CRC predictive ability. The panel of 22 viral markers was reported to improve the performance of a bacteria-based random forest classifier and FOBT or FIT tests for the detection of CRC. However, these markers failed to distinguish between adenomas and controls, similar to bacterial markers.

Together, these studies suggest that components of the gut microbiota other than bacteria could have the ability to distinguish between CRC patients and healthy individuals and require further exploration of their diagnostic capabilities in much larger patient cohorts.

### CRC prognostic and treatment stratification potential of gut microbiome

In addition to the diagnostic potential of gut dysbiosis in CRC, these microbial biomarkers may have prognostic potential, and as a stratification aid to select patient treatment and predict patient outcome. For example, increased presence of *F. nucleatum* is often associated with poorer prognosis and reduced survival after diagnosis, and patients with high *F. nucleatum* have been suggested to be more likely to have tumours displaying DNA microsatellite instability compared with patients with low *F. nucleatum* [50].

Experiments in CRC cell lines found that *F. nucleatum* infection reduced chemosensitivity and this has been attributed to an observed up-regulation of BIRC_3_ [44]. BIRC_3_ is a member of the inhibitor of apoptosis proteins (IAPs), and can inhibit the apoptotic caspase cascade [91]. Treatment response and the association with the microbiome has also been studied, and several papers have shown that the microbiota can alter responses to anticancer therapies both positively and negatively. For example, chemotherapy efficacy in *Caenorhabditis elegans*, is altered depending on its bacterial diet. *C. elegans* fed a *Comamonas* diet responded well (produced viable offspring rather than dead) to 5-fluoro-2′-deoxyuridine (FUDR) chemotherapy, but worse when exposed to camptothecin (CPT). In contrast *C. elegans* supplemented with *E. coli OP50* diet had the opposite effect and fared better with CPT than FUDR. However, there was no difference in 5-fluorouracil (5-FU) efficacy in *C. elegans* with either *Comamonas* or *E. coli OP50* diets [92]. Whilst the cause is not fully understood, several bacterial metabolic genes and pathways have been implicated such as those involved in nucleotide metabolism [92,93].

One pilot study by Shi et al., [94] (2020) looked at the response of rectal cancer patients to neoadjuvant chemoradiotherapy, and the associations with the gut microbiome. Patients were classified into responders and non-responders (10 and 12 patients respectively), and also patients who had diarrhoea vs non or mild diarrhoea (14 and 8 patients). Following 16S rRNA sequencing, *Shuttleworthia* was found to be enriched in responders, and several bacteria taxa in the Clostridiales order were found to be enriched in non-responders. Also, *Bifidobacterim, Clostridia* and *Bacterioides* were found to be enriched in patients with no, or mild diarrhoea. Whilst this study was only on a very small cohort, further investigation may fully validate these markers as being able to predict therapy response and toxicity.

Due to extreme variations in study findings, including the number and type of microbial markers, there is a need for larger scale analyses to be carried out to identify a potential consensus panel. Several studies and meta-analyses have been carried out to investigate the abilities of novel diagnostic models to be used on different cohorts, including cross-cohort comparisons across various geographical locations [48,95,96]. Whilst many studies have investigated the microbiome as diagnostic and prognostic markers for CRC, there are many barriers to these being introduced in the clinic. To date, there is no consensus in terms of the microbial alterations in CRC, but also on the markers that have the best prognostic or diagnostic value [97]. This is in part due to the complexity of the gut microbiome, but also other technical and biological parameters [97]. It is yet to be fully understood whether many bacteria or microbes described are directly involved in CRC carcinogenesis, or whether they are merely passengers. Additionally, many factors affect an individual’s microbiome, such as diet, age and stage of disease – all of which would affect the performance of microbiome-based models. Additionally, many studies have found varying results, which may be based on the techniques used (metagenomics or 16S rRNA next-generation sequencing). Even the taxonomic level considered and regions of the 16S rRNA sequenced may result in variation [97].

## Bacterial therapeutic approaches

**Figure 2:**
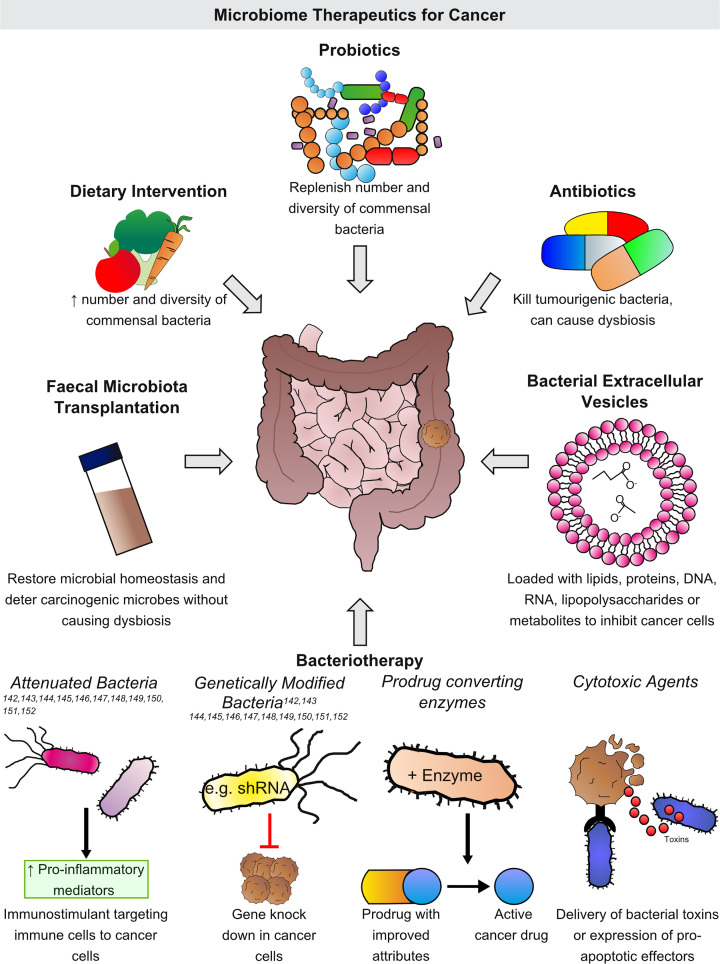
Harnessing the microbiome as therapeutic tools in the treatment of solid cancers The involvement of the gut microbiome and pathogenic bacteria in the development of cancer has been previously documented. Novel therapeutics strategies are being developed to modify the gut microbiome to aid cancer treatment. The gut microbiome can be modified through alteration to a person’s diet and lifestyle, the administration of probiotics, faecal microbiota transplantation or antibiotic therapies. More recently the use of targeted modulation using bacteria has emerged, known as bacteriotherapy. Importantly attenuated bacteria that specifically target the tumour are used to initiate an immune response without the undesirable toxicities usually associated with the bacterium. Such bacteria are often also genetically modified to improve their tumour targeting ability and to deliver various biomolecules, prodrug-converting enzymes or toxins to promote cancer cell death. Often clinical trials exploit multiples arms of these microbial therapies to improve tumour treatment and as such this highlights the flexibility of these therapeutic approaches.

The microbiome not only impacts tumorigenesis, but there is evidence demonstrating that the microbiota plays an important part in the host response to chemotherapy and immunotherapy [98]. Consequently, studies are ongoing to look at the effects of modulating the tumour microenvironment using antibiotics, probiotics and faecal microbiota transplants (FMTs) (Figure 2).

### Antibiotics and probiotics

The use of antibiotics to modulate the tumour microenvironment as a therapeutic agent against CRC is a controversial topic. On one hand, an antibiotic can be effectively employed to target tumorigenic bacteria. For example, as previously discussed, *F. nucleatum* has been shown to increase tumorigenesis and promote chemoresistance, especially to 5-FU in CRC [47,99]. Antibiotic treatment with metronidazole in *F. nucleatum* positive patient-derived xenograft (PDX) mouse models revealed a significant decrease in tumour cell proliferation and tumour growth with a reduction in *F. nucleatum* bacterial load [100].

However, it is widely argued that this is an overly aggressive and non-selective way to target these tumorigenic bacteria, often resulting in dysbiosis and could have adverse impact on further treatment. Multiple studies have found that germfree mice or mice pre-treated with antibiotics, do not respond as well to chemotherapy, such as oxaliplatin, or immunotherapy, such as antibodies targeting CTLA-4 (a suppressor of T-cell activation), in comparison to control mice with an intact microbiome. This effect is due to the disruption of the microbiota and its ability to modulate the tumour microenvironment and the innate immune system after treatment [101,102].

Instead, preliminary research is being carried out using probiotics as a prophylactic, to prevent CRC development. The most common probiotic bacteria currently in use are *Lactobacillus spp.* and *Bifidobacteria spp.* both of which are lactic acid bacteria. These anaerobic strains are found naturally in the microflora and as probiotics they provide many benefits to the intestinal gut health from maintenance of homoeostasis through to modulation of immune and inflammatory factors [103]. One clinical trial found that the level of circulating pro-inflammatory cytokines (such as TNFα and IL-10) in the serum was significantly reduced after treatment with a probiotic product compared with the placebo group. In this trial, 52 post-surgical stage III CRC patients, in a randomised double-blind placebo-controlled trial, were given either a placebo or a probiotic product containing 30x10^10^ [10] colony forming units (CFUs) of six viable *Lactobacillus* and *Bifidobacteria* strains, twice daily for 6 months [104]. This is important as an increased level of circulating pro-inflammatory cytokines has often been associated with poor overall survival in CRC patients [105].

Furthermore, probiotics can modulate the tumour microenvironment, encouraging microflora diversity and it has been suggested that probiotics could potentially reduce the levels of pathogenic/tumorigenic bacteria such as *F. nucleatum* spp. [106,107]. However, greater pre-clinical and clinical exploration to determine the effectiveness in using probiotics to aid the treatment of CRC is required.

###  FMT

Faecal microbiota transplantation is the transference of faecal microbes and other faecal materials from a healthy donor to an ailing patient. The aim of FMT is to permanently restore microbial homoeostasis and unlike antibiotics, FMT does not lead to dysbiosis. Ultimately, this leads to a reduction in complications resulting from treatment and can increase treatment effectiveness through immunotherapeutic effects and the determent of opportunistic, pathogenic bacteria associated with tumorigenesis. FMT first gained popularity due to its effectiveness at treating patients with recurring *Clostridium difficile* infections and was shown to be as well tolerated, yet more effective than antibiotics alone [108]. More recently, FMT has been shown to reverse the adverse side effect of chemotherapies, such as FOLFOX, in CRC mouse models, restoring the microbiota diversity, correcting dysbiosis and reducing the effect of chemotherapy-induced diarrhoea and intestinal mucositis [109,110]. However, large-scale clinical trials are required to fully establish the safety and efficacy of utilising FMT in cancer management before it can be fully employed in the clinic [111].

### Bacteriotherapy

Despite evidence that some species of bacteria contribute to tumorigenesis, the use of bacteria as a cancer therapeutic is not a novel concept and has been around since the late 1800s, where the technique was first trialled by William B. Coley, who is now considered to be the father of immunotherapy. However, despite his relative success at the time, the technique was overlooked until recent years, where interest has spiked in developing immunological approaches to cancer therapeutics. Leading to the publication of a ‘white paper on microbial anti-cancer therapy and prevention’ after the Microbial Based Cancer Therapy Meeting (2018) in an initial attempt to define microbial therapy [112]. Some bacterial species can be utilised to enhance immune responses with anticancer effects and this area of research has been named bacteriotherapy (Figure 2).

#### Attenuated bacteria

The majority of bacteria being investigated as anticancer agents are obligate or facultative anaerobic bacteria predominately *Clostridium* spp., *Escherichia coli* spp. or *Salmonella* spp. as these bacteria allow the selective colonisation of tumour tissues, taking advantage of the hard to reach, associated region of hypoxia and nutrient rich areas of necrosis [113]. Although the mechanism of the tumour-trophic nature of these bacteria is not always well defined, their anti-tumour effects have been well documented and vary dependent on the strain of bacteria used. Furthermore, these strains are usually attenuated, which decreases undesirable side effects maintaining safety to the host [114].

However, this attenuation requires balance, increasing the safety aspect for the host whilst maintaining the bacteria potential as an immunostimulant, which is one of the key mechanisms employed against the cancer. An example of where this balance is necessary is the bacterial flagellum. The bacterial flagellum aids in mobility of the bacteria, enabling the bacteria to move deep into the tumour tissue and has been shown to be necessary to increase anticancer effectiveness as a therapeutic [115,116]. However, it is also a potent virulence factor which has been determined to stimulate a host immune response as a PAMP which is recognised by TLR5 [117]. Upon this interaction, there is an increase in pro-inflammatory cytokines and a decrease in anti-inflammatory and angiogenic factors which leads to inhibition of the tumour growth.

Attenuated bacteria are already being used as effective standard of care cancer therapies in the clinic. One key example of this is in non-muscle-invasive bladder cancer (NMIBC). Patients at high risk of the cancer progressing beyond the lining of the bladder are offered treatment with a variant of the Bacillus Calmette-Guérin (BCG) vaccine, a live attenuated strain of *Mycobacterium bovis*. The vaccine is administered via a catheter into the bladder and stimulates an immune response which continues to target the tumour cells after it has removed the pathogen [118,119]. It has been established that the vaccine not only initiates the recruitment of immune cells to the area, but also encourages the cancer cells to produce cytokines, such as IL-8, further highlighting themselves to the immune cells [119].

However, this immunostimulatory effect is not the only mechanism exploited utilising attenuated bacteria. Extracellular bacterial products, such as bacteriocins, toxins and bacterial peptides, are also being investigated for their therapeutic potential. Bacteriocins are ribosomally synthesised antimicrobial peptides produced by bacteria to kill or inhibit the growth of competing microorganisms and were first discovered by Gratia in 1925. This led to the discovery of bacteriophages and the development of antibiotics. Bacteriocins are protein-based toxins. There are multiple different methods to classify them varying from classifications based on killing methods, genetics or more commonly the molecular weight [120]. Bacteriocins have been shown to have a variety of anticancer effects such as membrane permeabilisation through pore formation, inhibiting tumour cell proliferation and inducing apoptosis, dependent on the bacteriocin used [121,122]. The use of bacteriocins, bacterial toxins and peptides as anti-cancer therapies in GI cancers are summarised in Soleimanpour et al., 2020 [123].

#### Genetically modified bacteria/delivery systems (plasmids/cassettes) – bactofection

Lately there has been an increased interest in genetically modified strains of bacteria to improve localisation to the tumour-site avoiding colonisation in the liver and spleen and to reduce toxicities. One such example is the modified attenuated *Salmonella entericia ser. typhimurium* (VNP20009) which has a deletion in the *msbB* and *purI* genes which reduce the LPS-associated toxicity of the bacterium and gave the bacterium a purine auxotrophic mutation, requiring the presence of adenine to survive [124]. VNP20009 has well-documented localisation in tumour tissue compared with adjacent tissue and well-characterised safety profiles in preclinical and clinical models [124]. VNP20009 was the first genetically modified bacterium to be utilised in a clinical trial, where it was demonstrated to be well tolerated below the maximum-tolerated dose (MTD) [125]. Furthermore, VNP20009 reportedly has a tumour-targeting nature in multiple cancer types, yet is not reported to promote tumour regression, consequently it is an ideal model to investigate its use as a delivery system for small molecules such as shRNA, antibodies, cytotoxic agents and prodrugs [113]. As such, this strain of *S. typhimurium* has been the base for many genetically modified and enhanced therapeutic strains. For example, the immune checkpoint protein indoleamine 2,3-dioxygenase 1 (IDO) is overexpressed in many CRC patients and has been linked to tumour mediated immunosuppression and results in a poor prognosis. In a study by Phan et al., (2020) attenuated *S. typhimurium* was used to deliver an shRNA plasmid against IDO (shIDO-ST) in two CRC murine models and was shown to effectively decrease the level of IDO and mitigate tumour growth in immunocompetent CRC mouse models compared with a scrambled shRNA control and a clinically relevant IDO inhibitor [126]. Furthermore, although not necessary in CRC models, this shIDO-ST therapy was effectively combined with immune checkpoint blockade (anti-PD-1/CTLA-4 antibodies) to enhance treatment efficacy in murine models of non-small cell lung cancer [127].

Promising commercial enterprises are now piloting bacterial delivery of specific therapeutic payloads to the lower GI tract in pre-clinical models. CHAIN Biotechnology Ltd (CHAIN Biotech) have developed a *Clostridium* Assisted Drug Development (CADD™) platform that utilises highly robust bacterial endospores (spores) formulated into capsules for orally delivered medicines that are both targeted and safe, with the additional benefit of being cost effective. These spores are resistant to the hosts stomach acid, bile acid and enzymatic digestion in the upper GI tract thus enabling them to reach the terminal ileum and large intestine where they can germinate and multiply. The growing cells are able to produce the bioactive of interest (e.g. metabolites, enzymes, or peptides) during transit through the lower GI tract. CHAIN’s host strain (*Clostridium*) which is naturally found in the gut, does not permanently colonise the human bowel offering a high degree of control over any therapeutic dosage. In addition to delivery of specific therapeutic agents, this differentiated *Clostridium* strain is able to secrete metabolites that have key roles in gut and immune system homoeostasis and thus could have the potential to reverse cancer gut dysbiosis. Whilst this cutting-edge research holds great promise for cancer therapeutics it is currently compounded by the requirement to prove that the delivery of live genetically modified organisms is completely safe to the patient and the wider community and the necessity to identify its full mechanism of action. The next challenge is taking CADD™ to clinical trials to demonstrate safety and efficacy in humans [128,129].

#### Prodrug-converting enzymes

Genetically modified bacteria can also be beneficial to cancer treatment by producing localised prodrug-converting enzymes which can metabolise systemic prodrugs and locally convert them into their active/cytotoxic forms. This offers the advantage of being able to improve the bioavailability of the drug as often the small molecules are able to penetrate further into the tumour and better interact with the cells. Furthermore, it can also decrease harmful off-target side effects. This technique is currently being investigated for multiple prodrugs using primarily *Salmonella spp.* and *Clostridium spp.* Most notably, for the use against CRC, the *S. typhimurium* VNP20009, mentioned previously, was further modified to express *E. coli* cytosine deaminase and named TAPET-CD [130]. TAPET-CD was utilised to convert the less toxic prodrug, 5-fluorocytosine (5-FC) into 5-FU, which then is converted into its toxic metabolites (5-dUMP, 5-FUTP and 5-FdUTP) intracellularly blocking thymidylate synthase activity and inducing apoptosis. TAPET-CD was shown to inhibit tumour growth in xenograft mouse models of CRC [130] and in a pilot clinical trial, these bacteria were found to colonise the intra-tumour microenvironment in two out of the three patients and increase the tumour localised conversion of 5-FU [131]. Patients for this pilot study were eligible if they had treatment-refractory advanced and/or metastatic solid tumours and in this case, all three had various head and neck cancers [131]. Moving forward, it is important to note that the efficacy of these systems is reliant on the localisation and sustainability of the bacteria providing continued prodrug-converting enzyme.

#### Cytotoxic agents

Another effector system developed for the use with tumour targeting bacteria is the delivery of cytotoxic agents. These could include bacterial toxins or the alternative immunotoxins; which are able to target specific cells through the connection of a ligand or antibody. This increases the specificity of the treatment and reduces toxicity, for example, a mutant strain of *S. typhimurium* was engineered to express an immunotoxin TGFα-PE38. This immunotoxin contained a ligand for epidermal growth factor receptor (EGFR), transforming growth factor α (TGFα), and a mutated *Pseudomonas* exotoxin A obtained from *Pseudomonas aeruginosa*, (PE38). The modified *Salmonella*, was used against mouse models of EGFR-positive colon and breast cancer to effectively target the tumour microenvironment and selectively kill the EGFR-expressing tumour cells, inhibiting further tumour growth [132]. However, bacterial toxins are not the only cytotoxic agent able to be utilised via this effector system. Recently researchers have also been looking into using tumour-targeting bacteria to deliver and express recombinant apoptosis-inducing ligands (such as TNFα, TRAIL or FASL). The use of this effector system is necessary to improve safety, avoiding the toxicities associated from direct use of the death ligands and also overcomes the challenges associated with the short half-life of these ligands. Multiple groups have successfully incorporated various plasmids of cDNA for human soluble tumour necrosis factor-related apoptosis-inducing ligand (TRAIL) into an attenuated strain of *Lactococcus lactis* to secrete biologically active TRAIL, which have proved to induce apoptosis in multiple human CRC cell lines including HCT116 and SW480 [133,134]. However, further research, including the use of *in vivo* models is still needed.

### Exploitation of BEVs as therapeutic agents

Previously we have discussed BEVs as potential biomarkers but their ability to carry effector molecules into host cells offers improvements in therapeutic delivery. For example, human EVs, commonly referred to as exosomes, are also altered in CRC, and play a role in its progression [135]. These EVs are nanosized lipid-encapsulated extracellular vesicles that contain lipids, proteins, DNA, RNA, LPSs and metabolites which facilitate intercellular interactions [136]. Emerging evidence suggests biomolecules loaded into EVs are transported to cancer or stromal cells, and can modulate signalling [137]. For example EVs secreted by the LIM1215 CRC cell line contain secreted mutant β-catenin that enhances activation of the Wnt pathway, a common alteration in CRC, in recipient RKO CRC cells despite them being wild type for β-catenin [138]. Whilst BEVs do not carry mutant β-catenin and thus would not contribute to tumorigenesis through this mechanism recent studies have set out to isolate BEVs from stool samples of cancer patients to identify their potential as cancer biomarkers [48,59] and delivery vehicles. Several *Bifidobacterium* and *Lactobacillus* strains have been shown to produce BEVs carrying molecules that are associated with the bacteria’s probiotic effects [139–141]. BEVs have been shown to alleviate food allergy response in murine models (*B longum* KACC 91563), have cytotoxic effects on HepG2 hepatic cancer cells (*Lactobacillus rhamnosus* GG) and protect the host from pathogenic bacteria (*L. plantarum*). Crucially effects were observed with EVs but not with whole bacterial cells, this is possibly due to the ability of EVs to penetrate the intestinal epithelial barrier, migrate to the other organs and interact with the host immune system more efficiently then complete bacteria [67,139,140]. Due to the composition and deliverability of compounds within EVs, repurposing of BEVs into clinical settings is gaining attention, including the delivery of antimicrobial compounds, reinforcing phage therapy (to overcome antibiotic resistance), vaccination and delivering health-promoting probiotics or food supplements, in addition to being diagnostic tools [67].

### Clinical trials

There are relatively few genetically engineered/attenuated bacteria currently in clinical trials to date, despite, as previously discussed, the first clinical trial involving the *S. typhimurium* VNP20009 strain occurring over 10 years ago in 1999 [125]. Yet this strain along with *Clostridium novyi-NT* and *Listeria monoctogenes* continue to be the most common strains of bacteria involved in clinical trials for solid tumour malignancies and CRC (Table 2). Despite the limited number of clinical trials, these Phase I trials have highlighted the importance of selecting the right dose and delivery method, to ensure the treatment is well tolerated and effective. Although the primary aim of these studies was to determine the MTD for each treatment, some early studies have published promising results indicating the potential of this treatment for future development [142].

**Table 2:** On-going and completed clinical trials against advanced solid tumour malignancies and CRC

Bacterial strain	Trial status	Phase	Cancer type	Delivery method	Number of patients	NCT/Reference
***S. typhimurium* VNP20009**	Completed	I	Treatment-Refractory advanced or metastatic solid tumours	Intratumoural	Not provided	NCT00004216 [143]
***S. typhimurium* VNP20009**	Completed	I	Advanced solid tumours	Intravenous	Not provided	NCT00006254 [144]
***S. typhimurium* VNP20009**	Completed	I	Treatment-Refractory advanced or metastatic cancer	Intravenous	45	NCT00004988 [145]
***S. typhimurium* VNP20009 expressing IL-2**	Completed	I	Patients with unresectable hepatic metastasis from a solid tumour	Oral	22	NCT01099631 [146]
***Clostridium novyi-NT***	Completed	I	Treatment-Refractory Solid Tumours	Intratumoural	24	NCT01924689 [147] Results published [142]
***Clostridium novyi-NT with Pembrolizumab***	Recruiting	Ib	Treatment-Refractory Solid Tumours	Intratumoural	18	NCT03435952 [148]
***Clostridium novyi-NT***	Terminated	I	Treatment-Refractory Solid tumours	Intravenous	5	NCT01118819 [149]
***Clostridium novyi-NT***	Terminated	II	Treatment-Refractory Solid tumours	Intravenous	2	NCT00358397 [150]
***Listeria monocytogenes (CRS-100)***	Completed	I	Adults with carcinoma and liver metastasis	Intravenous	9	NCT00327652 [151]
***Listeria monocytogenes (pLADD)***	Terminated	I	Microsatellite stable metastatic CRC	Intravenous	28	NCT03189030 [152]

## Conclusion

Due to extreme variations in study findings, including the number and type of microbial markers, there is a need for larger scale analyses to be carried out to identify a potential consensus CRC microbial panel. With several studies and meta-analyses having been carried out to investigate the abilities of novel diagnostic models to be used on different cohorts, including cross-cohort comparisons across various geographical locations [45,92,93]. However, despite the promising nature of many of the approaches described here a recurring theme is the preliminary nature of the work carried out to date and the requirement for larger validation studies, mechanistic understanding and safety profiling. With cancer incidence and morbidity increasing globally, funders and policy makers are recognising that the economic burden of funding and delivering curative research can be significantly reduced by improving approaches to prevention and early detection. We are each defined by our genetic, epigenetic, microbial and lifestyle behaviours. To deliver a world where people are less likely to develop or succumb to cancer requires personalised prevention and improved early detection approaches. To achieve this will require multifaceted whole person approaches that encompass all tools at our disposal; from education to improving lifestyle behaviours, provision of healthy dietary components, a better understanding of personal genetic, epigenetic and microbial risks to state-of-the-art technology for detecting indicators of poor health or early disease. To address these, we require funding support for high-risk longitudinal studies that improve our understanding of lifelong health and permit studies that seek to definitively prove the efficacy of preventive and chemo-preventative approaches. The latest aspirin trials on the CaPP3 – Colorectal Adenoma Carcinoma Prevention Programme 3 (ccapp3.org) are a fine exemplar of this approach, where opportunities are available for small window trials to occur within this large body of work.

## Open Access

Open access for this article was enabled by the participation of Cardiff University in an all-inclusive *Read & Publish* pilot with Portland Press and the Biochemical Society under a transformative agreement with JISC.

## Abbreviations

AUC: area under the curve
BEV: bacterial extracellular vesicle
CPT: camptothecin
CRA: colorectal adenoma
CRC: colorectal cancer
EGFR: epidermal growth factor receptor
FIT: faecal immunochemical test
FMT: faecal microbiota transplantation
FOBT: faecal occult blood test
FUDR: 5-fluoro-2′-deoxyuridine
GI: gastrointestinal
IDO: indoleamine 2,3-dioxygenase 1
LPS: lipopolysaccharide
MTD: maximum-tolerated dose
PAMP: pathogen-associated molecular pattern
SCFA: short-chain fatty acid
TLR: Toll-like receptor
TRAIL: tumour necrosis factor-related apoptosis-inducing ligand
VOC: volatile organic compound
5-FU: 5-fluorouracil
